# Robinsoniella Bacteremia in a Patient With Radiation Proctitis and Prior Colon Cancer: A Rare Case

**DOI:** 10.7759/cureus.115958

**Published:** 2026-09-08

**Authors:** Alexa Markl, Joshua Iskander, Ivan Hutchison, Simeon Mihaylov, Jeffrey Lach

**Affiliations:** 1 Internal Medicine, Lake Erie College of Osteopathic Medicine, Erie, USA; 2 Internal Medicine, University at Buffalo, State University of New York, Buffalo, USA; 3 Internal Medicine, Catholic Health, Buffalo, USA

**Keywords:** anaerobic bacteremia, bacteremia, immunocompromised, radiation proctitis, robinsoniella

## Abstract

*Robinsoniella* species are strictly anaerobic, Gram-positive, spore-forming bacilli infrequently associated with human bloodstream infections. We present a case of a 49-year-old woman with a history of colon and anal carcinoma treated with radiation and chemotherapy. She presented with fever, dyspnea, and worsening diarrhea. Blood cultures grew *Serratia marcescens*, *Robinsoniella* species, and *Clostridium* species, and she was initially treated with intravenous piperacillin-tazobactam and vancomycin, followed by cefepime, before ultimately transitioning to ertapenem. Although no treatment is currently established for *Robinsoniella peoriensis*, evidence remains insufficient to define an optimal antimicrobial regimen. This case highlights the pathogenic potential of *Robinsoniella* species in immunocompromised patients and underscores the importance of considering gastrointestinal pathology as a source of bacteremia involving this uncommon anaerobic organism.

## Introduction

*Robinsoniella peoriensis* (*R. peoriensis*) is a rare anaerobic, Gram-positive, spore-forming bacillus and is exceedingly rare in human infections [1,2]. The organism can be difficult to identify using conventional microbiological methods, and advances in molecular diagnostics, including 16S rRNA gene sequencing and matrix-assisted laser desorption/ionization time-of-flight mass spectrometry, have largely facilitated its recognition [2,3]. Consequently, the true incidence of *R. peoriensis* infections may be underestimated, and the clinical significance of this pathogen remains incompletely understood.

Human infections attributed to *R. peoriensis* have been reported only sporadically and encompass a broad clinical spectrum, including osteoarticular infections, peritonitis, infective endocarditis, surgical site infections, and bacteremia [4]. Bloodstream infections appear to occur predominantly in patients with significant underlying comorbidities, such as malignancy, recent surgical interventions, or immunocompromised states [2]. One of the earliest reported cases involved bacteremia in a patient with pancreatic cancer, highlighting the organism's potential to cause invasive disease in susceptible hosts [3]. More recently, cases of bacteremia occurring despite broad-spectrum antimicrobial therapy and reports of severe sepsis with fatal outcomes have further emphasized the pathogenic potential of this uncommon organism [1,2]. Antimicrobial susceptibility in *R. peoriensis* has varied among the few reported cases, and no standard therapy is currently established for this rare organism. Published reports describe treatment failure with some penicillin-based regimens and favorable responses to carbapenems, specifically meropenem, ertapenem, or imipenem, although this remains under investigation [1].

Given the limited number of published cases, significant gaps remain in the epidemiology, antimicrobial susceptibility patterns, optimal treatment strategies, and outcomes of *R. peoriensis* bacteremia. The following case illustrates the diagnostic and therapeutic challenges associated with this organism and highlights the importance of considering *Robinsoniella *species as potential pathogens in susceptible patients. This case report describes a 49-year-old woman with *Robinsoniella* bacteremia, subsequently treated with ertapenem.

## Case presentation

A 49-year-old woman with a complex medical history significant for end-stage renal disease (ESRD) secondary to IgA nephropathy on maintenance hemodialysis four times weekly after two failed renal transplants, heart failure with preserved ejection fraction, coronary artery disease, paroxysmal atrial fibrillation currently on apixaban, chronic hypotension requiring midodrine, and previous colon and anal carcinoma treated with chemotherapy and pelvic radiation was admitted for five days of fever >101°F, dyspnea, non-localized abdominal pain, and acute on chronic bloody diarrhea. She was four years status post chemotherapy, pelvic radiation, and transverse colectomy for colon and anal carcinoma. Vital signs on presentation included a temperature of 101.1°F, heart rate of 94 beats/min, blood pressure of 90/48 mmHg, and respiratory rate of 21 breaths/min. In the setting of suspected infection, she met the criteria for severe sepsis. Table 1 presents the initial laboratory findings.

**Table 1:** Patient lab values on hospital day 1 WBC: white blood cell, PT: prothrombin time, INR: international normalized ratio

Lab	WBC	Hemoglobin	Platelets	Sodium	Potassium	Creatinine	Anion gap	Bicarb	PT	INR
Value	3.8	10.4	119	137	4.4	5.9	8	28	17.9	1.6

Further infectious workup included blood cultures, chest X-ray, CT of the abdomen and pelvis, viral panels, and methicillin-resistant *Staphylococcus aureus* (MRSA) nasal swabs. A chest X-ray (Figure 1) revealed severe pulmonary vascular congestion and small bilateral pleural effusions; pneumonia could not be excluded. Viral panels were negative. CT of the pelvis (Figure 2), lower extremity ultrasonography, and radionuclide bone scanning demonstrated diffuse soft tissue edema but no drainable abscess, gas formation, osteomyelitis, calciphylaxis, soft tissue calcification, or deep venous thrombosis. Blood cultures showed mixed Gram-positive/Gram-negative bacteremia, with growth of *Serratia marcescens*, *Robinsoniella* species, and *Clostridium* species. Susceptibilities are listed below for *Serratia marcescens* (Table 2). Stool samples and *Clostridium difficile* testing were negative.

**Figure 1:**
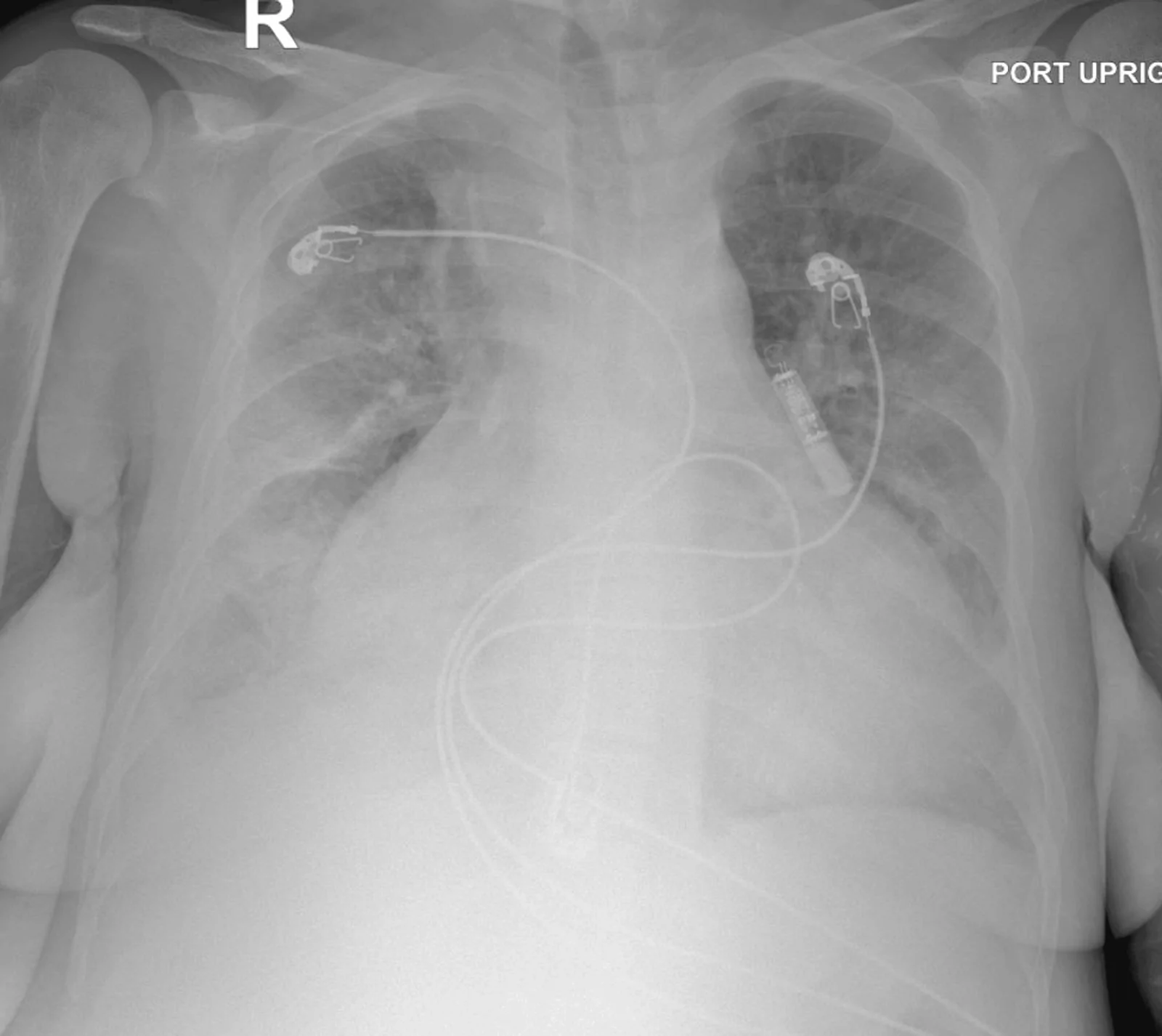
AP chest X-ray obtained at the time of presentation revealed a massively enlarged cardiac silhouette and an implanted cardiac device. Probable severe pulmonary vascular congestion and small bilateral pleural effusions were noted. Pneumonia could not be excluded. No pneumothorax was identified AP: anteroposterior

**Figure 2:**
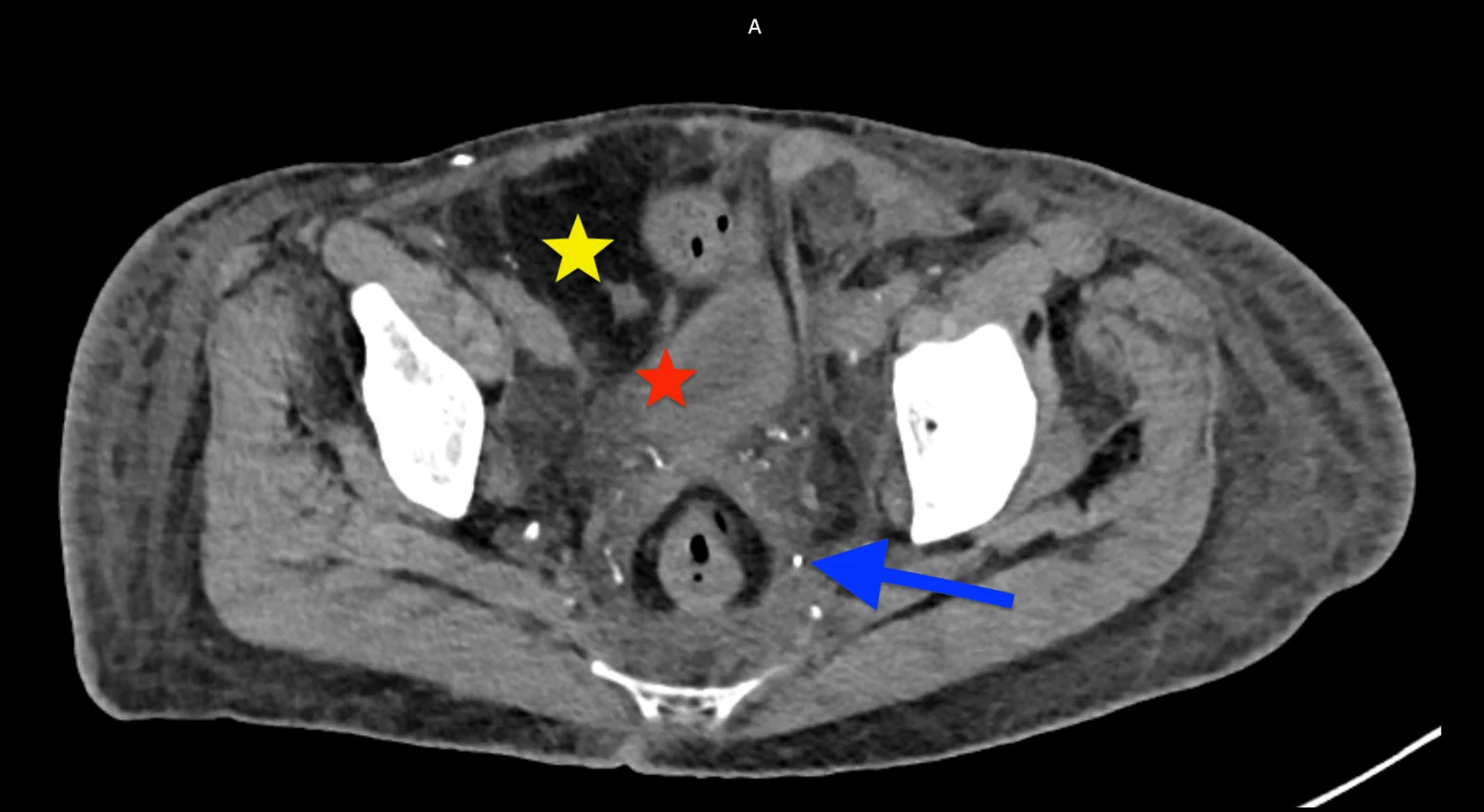
CT of the abdomen and pelvis without contrast revealed severe pelvic stranding with bladder wall thickening and surrounding inflammatory changes. Extensive vascular calcifications and pelvic phleboliths were also noted. The uterus was surgically absent. No evidence of an abscess or identifiable source of infection was observed The red star indicates bladder wall thickening. The yellow star indicates hazy pelvic stranding and inflammatory changes. The blue arrow indicates extensive vascular calcifications and pelvic phleboliths. CT: computed tomography

**Table 2:** Antimicrobial susceptibility profile of Serratia marcescens MIC: minimal inhibitory concentration

*Serratia marcescens* - MIC	
Drug (s)	Susceptibility
Ampicillin	Resistant
Ampicillin-sulbactam	Resistant
Cefazolin	Resistant
Cefepime	Susceptible
Ceftriaxone	Resistant
Ertapenem	Susceptible
Gentamicin	Susceptible
Levofloxacin	Susceptible
Piperacillin-tazobactam	Resistant
Trimethoprim-sulfamethoxazole	Susceptible

Due to her unknown source of infection and possible pneumonia, she was initially treated empirically with intravenous piperacillin-tazobactam and vancomycin on day 1 of admission. MRSA nasal swabs were negative on day 2 of admission, and vancomycin was discontinued. Cefepime was initiated on day 2 for broader antimicrobial coverage after four blood cultures grew Gram-negative organisms. On day 4, blood cultures grew *Robinsoniella* and *Serratia*,* *and infectious disease recommended transitioning from cefepime to ertapenem. Her gastrointestinal symptoms began to improve after initiation of ertapenem, supporting *Robinsoniella* as a potential contributor to the bacteremia. Her respiratory symptoms continued to improve with supplemental oxygen and antimicrobial therapy.

By day 4 of her hospital course, the source of bacteremia was still unknown. The patient continued to have pitting edema and pain involving the left thigh and buttock. There were no open wounds or areas of fluctuance or purulent drainage. Repeat imaging obtained on hospital day 5 remained negative for infectious myositis or other focal pathology.

Polymerase chain reaction was performed on a stool sample. It was negative for *Shigella*, *Salmonella*, *Campylobacter*, Shiga toxin-producing *Escherichia coli*, *Plesiomonas shigelloides*, *Vibrio*, *Yersinia*, enterotoxigenic *Escherichia coli*, *Giardia*, *Cryptosporidium*, and *Entamoeba*. The comprehensive ova and parasite examination was negative. Fecal fat and celiac panels were unrevealing. Strongyloides IgG antibody testing was performed because helminth infections may be associated with recurrent enteric bacteremia; the result was negative. Colonoscopy was performed to evaluate chronic diarrhea along with acute rectal bleeding. Examination revealed radiation proctitis without evidence of recurrent malignancy, inflammatory bowel disease, or colonic masses. These findings suggested that radiation proctitis was the most likely source of her bacteremia. Canasa suppositories were recommended for ongoing management of radiation proctitis, and the patient's pain was managed conservatively with opioid analgesics. She was also advised to continue her five-day course of ertapenem for her bacteremia.

Regarding her ESRD, the patient continued regular hemodialysis throughout hospitalization with ultrafiltration for volume management. Imaging also demonstrated markedly atrophic native kidneys with extensive vascular calcifications consistent with chronic ESRD. The patient also presented with chronic pancytopenia, which was attributed to prior chemotherapy and radiation exposure, and an outpatient bone marrow biopsy was arranged for further evaluation. Over the course of treatment, the patient gradually improved clinically and was discharged with a peripherally inserted central catheter to complete intravenous ertapenem therapy, along with a seven-day course of oral trimethoprim-sulfamethoxazole and metronidazole for continued polymicrobial infection coverage. Given the suspected gastrointestinal source and polymicrobial bacteremia, coverage of both aerobic and anaerobic organisms was considered warranted in addition to ertapenem therapy.

## Discussion

*R. peoriensis* remains an exceptionally rare cause of human infection, with only 20-25 documented cases worldwide, including only seven documented cases of bacteremia (Table 3). Most reported cases involve patients with impaired immunity, malignancy, chronic wounds, prosthetic material, or gastrointestinal pathology [4]. Because the organism is an obligate anaerobe and difficult to identify using conventional laboratory methods, the true incidence is likely underestimated [3,4].

**Table 3:** Documented cases of R. peoriensis bacteremia reported in the literature AMPC: amoxicillin, DOXY: doxycycline, IDC: idiopathic dilated cardiomyopathy, IMP: imipenem, LVFX: levofloxacin, MEPM: meropenem, MNZ: metronidazole, PIPC/TAZ: piperacillin-tazobactam, ST: trimethoprim-sulfamethoxazole, VCM: vancomycin, ETP: ertapenem, *R. peoriensis*: *Robinsoniella peoriensis*

Case/reference	Age	Sex	Primary disease	Immunocompromising factor	Portal of entry	Treatment	Duration of treatment	Confirmation of negative blood culture	Outcome
Ursenbach et al., 2020 [5]	79	Female	IDC	No	Unknown	AMPC, DOXY	6 weeks	Yes	Improved
Jeon et al., 2012 [6]	76	Male	Stroke	No	Unknown	MEPM, VCM, ST	Unknown	Yes	Died (primary disease)
Lim et al., 2017 [7]	63	Male	Stroke, lung cancer	Chemotherapy	Unknown	IMP	Unknown	Yes	Died (unknown)
Gomez et al., 2011 [8]	79	Female	Stroke, lung cancer	No	Unknown	PIPC/TAZ, LVFX, MNZ	4 days	No	Died (bacteremia)
Shen et al., 2010 [3]	42	Male	Pancreatic cancer	Chemotherapy	Intestinal mucosa	MNZ	Unknown	Yes	Died (primary disease)
Mejia-Gomez et al., 2022 [9]	47	Female	Small bowel cancer, uterine cancer	Chemotherapy	Intestinal mucosa	PIPC/TAZ, DOXY	2 weeks	Yes	Died (primary disease)
Furuya et al., 2023 [1]	84	Female	Peritoneal cancer	Chemotherapy	Intestinal mucosa	MEPM, VCM	16 days	Yes	Improved
Present case	49	Female	Colon and anal cancer	Chemotherapy	Rectal mucosa	ETP	7 days	No	Improved

This patient's presentation supports an enteric source as the likely source of infection, although pneumonia represents a possible competing source. Multiple factors may have contributed to the disruption of mucosal barrier integrity, including chronic radiation proctitis, persistent diarrhea, previous colorectal malignancy, and immunosuppression associated with ESRD and prior renal transplantation. The simultaneous isolation of *Serratia*, *Clostridium*, and *Robinsoniella* further supports an enteric source rather than isolated contamination [2].

Patients with ESRD have impaired innate and adaptive immune function, placing them at increased risk for bloodstream infections [4]. Previous chemotherapy and radiation likely compounded immune dysfunction, while chronic radiation injury may have provided a persistent portal of entry for bacteria [2,3]. Although extensive imaging excluded abscess, osteomyelitis, and soft tissue infection, polymicrobial bacteremia without another source strongly implicates gastrointestinal mucosal disruption [2].

No standardized antimicrobial recommendations exist because of the limited number of reported cases. Published isolates generally demonstrate susceptibility to beta-lactam/beta-lactamase inhibitor combinations, carbapenems, and metronidazole, although susceptibility testing remains important given the limited available data [1]. Our patient's clinical response following carbapenem therapy further supports the effectiveness of broad anaerobic coverage in severe infections involving *Robinsoniella* [1]. Recognition of *Robinsoniella* should prompt evaluation for underlying gastrointestinal pathology, especially in immunocompromised patients with polymicrobial bloodstream infections.

## Conclusions

*Robinsoniella* species, although rarely seen in human infections, can cause severe bacteremia, particularly in immunocompromised patients. This case report highlights important considerations regarding the clinical presentation, diagnosis, antimicrobial susceptibility, and treatment of *Robinsoniella* infections. Although treatment failure with piperacillin-tazobactam has been reported, carbapenems have demonstrated favorable clinical activity in several reported cases. Antimicrobial selection should be individualized according to susceptibility results when available. Clinical outcomes and disease progression depend heavily on the patient's immunocompromised state and the type of infection. Given the rarity of reported infections, future investigations are essential to improve understanding of the clinical spectrum, epidemiology, and outcomes associated with *Robinsoniella* species, while also clarifying the molecular mechanisms and genetic factors that contribute to antimicrobial resistance.

## References

[1] Furuya K, Ito K, Sugiyama K, Makio S, Shimada T (2023). A case of Robinsoniella peoriensis bacteremia during using piperacillin-tazobactam. J Infect Chemother.

[2] Huang M, Zhang H, Li X, Zhang X, Xu Y (2026). A fatal case report of sepsis caused by Robinsoniella sp. Rev Inst Med Trop Sao Paulo.

[3] Shen D, Chen R, Ye L, Luo Y, Tang YW (2010). Robinsoniella peoriensis bacteremia in a patient with pancreatic cancer. J Clin Microbiol.

[4] Ioannou P, Baliou S, Kofteridis D (2024). Robinsoniella peoriensis infections in humans-a narrative review. Antibiotics (Basel).

[5] Ursenbach A, Ruch Y, Von Hunolstein JJ, Lefebvre N, Argemi X, Hansmann Y, Schramm F (2020). First case of Robinsoniella peoriensis endocarditis. Med Mal Infect.

[6] Jeon Y, Kim TS, Kim HB, Park KU, Song J, Kim EC (2012). First Korean case of Robinsoniella peoriensis bacteremia in a patient with aspiration pneumonia. Ann Lab Med.

[7] Lim S, Huh HJ, Lee NY (2017). Robinsoniella peoriensis bacteremia: a second case in Korea. Ann Lab Med.

[8] Gomez E, Gustafson DR, Colgrove R, Ly T, Santana R, Rosenblatt JE, Patel R (2011). Isolation of Robinsoniella peoriensis from four human specimens. J Clin Microbiol.

[9] Mejia-Gomez J, Zigras T, Patel Y (2022). First reported case of Robinsoniella peoriensis pyometra and bloodstream infection: a case report and review of the literature. Anaerobe.

